# Characterization of HIV-1 Infection in Microglia-Containing Human Cerebral Organoids

**DOI:** 10.3390/v14040829

**Published:** 2022-04-16

**Authors:** Stephanie B. H. Gumbs, Amber Berdenis van Berlekom, Raphael Kübler, Pauline J. Schipper, Lavina Gharu, Marco P. Boks, Paul R. Ormel, Annemarie M. J. Wensing, Lot D. de Witte, Monique Nijhuis

**Affiliations:** 1 Translational Virology, Department of Medical Microbiology, University Medical Center Utrecht, 3584 CX Utrecht, The Netherlands; s.b.h.gumbs@umcutrecht.nl (S.B.H.G.); raphael.kubler@mssm.edu (R.K.); p.j.schipper@umcutrecht.nl (P.J.S.); l.gharu@umcutrecht.nl (L.G.); a.m.j.wensing@umcutrecht.nl (A.M.J.W.); 2 Department of Psychiatry, University Medical Center Utrect Brain Center, Utrecht University, 3584 CG Utrecht, The Netherlands; a.berdenisvanberlekom-3@umcutrecht.nl (A.B.v.B.); m.p.m.boks@umcutrecht.nl (M.P.B.); p.ormel@gmail.com (P.R.O.); lotje.dewitte@mssm.edu (L.D.d.W.); 3 Department of Translational Neuroscience, University Medical Center Utrecht Brain Center, Utrecht University, 3584 CG Utrecht, The Netherlands; 4 Department of Psychiatry, Icahn School of Medicine at Mount Sinai, New York, NY 10029, USA

**Keywords:** microglia, HIV, HIV-associated neurocognitive disorder, neuropathogenesis, central nervous system, organoid, matrigel

## Abstract

The achievement of an HIV cure is dependent on the eradication or permanent silencing of HIV-latent viral reservoirs, including the understudied central nervous system (CNS) reservoir. This requires a deep understanding of the molecular mechanisms of HIV’s entry into the CNS, latency establishment, persistence, and reversal. Therefore, representative CNS culture models that reflect the intercellular dynamics and pathophysiology of the human brain are urgently needed in order to study the CNS viral reservoir and HIV-induced neuropathogenesis. In this study, we characterized a human cerebral organoid model in which microglia grow intrinsically as a CNS culture model to study HIV infection in the CNS. We demonstrated that both cerebral organoids and isolated organoid-derived microglia (oMG), infected with replication-competent HIVbal reporter viruses, support productive HIV infection via the CCR5 co-receptor. Productive HIV infection was only observed in microglial cells. Fluorescence analysis revealed microglia as the only HIV target cell. Susceptibility to HIV infection was dependent on the co-expression of microglia-specific markers and the CD4 and CCR5 HIV receptors. Altogether, this model will be a valuable tool within the HIV research community to study HIV–CNS interactions, the underlying mechanisms of HIV-associated neurological disorders (HAND), and the efficacy of new therapeutic and curative strategies on the CNS viral reservoir.

## 1. Introduction

HIV enters the central nervous system (CNS) early during infection mainly through infected monocytes or CD4+ T lymphocytes and, to a lesser extent, as viral particles crossing the blood–brain barrier (BBB) [1,2,3]. Despite this modern era of antiretroviral therapy (ART), characterized by the suppression of HIV replication, roughly 50% of treated HIV-infected individuals are afflicted with a range of cognitive impairments, collectively termed HIV-associated neurocognitive disorders (HAND) [4,5]. The onset and progression of HAND are still unknown but hypothesized to be multifactorial, including continued immune dysregulation and residual chronic inflammation in response to viral persistence and production, and the ensuing accumulation of cytotoxic viral proteins [6,7,8].

Once it crosses the BBB, HIV mainly infects microglia and, to a lesser extent, macrophages that express both the CD4 and the CCR5 co-receptors required for productive HIV infection [9]. HIV DNA and/or RNA have been detected in 1–10% of microglia and macrophages in both untreated and virally suppressed individuals who died with and without (severe) HAND [10,11,12,13,14]. HIV DNA has also been detected in the astrocytes (0.4–5.2%) of virally suppressed individuals; however, whether astrocytes support productive HIV infection remains controversial, as they do not express the CD4 receptor [15,16]. Instead, HIV entry into astrocytes is proposed to occur via receptor-mediated endocytosis or direct cell-to-cell contact with CD4+ infected T-cells [16,17,18,19,20]. Neurons, however, are presumed to be overall resistant to HIV infection. Furthermore, microglia and, to a lesser extent, astrocytes have a long lifespan and can undergo cell division, which enables them to function as a stable, long-term HIV reservoir in the CNS [16,21,22].

To achieve an HIV cure, we need to eradicate or permanently silence all HIV viral reservoirs, including the CNS. Hence, we need a thorough understanding of the molecular mechanisms of HIV CNS entry, latency establishment, viral persistence, HIV-induced neuropathogenesis, and reactivation of the latent virus. However, due to ethical and technical restrictions, neuroHIV research has been predominantly confined to the examination of postmortem brain tissue and two-dimensional (2D) CNS culture models, such as primary cultures from human brain tissues and in vitro-differentiated CNS cells [23,24,25,26,27]. Alternatively, non-human primates (NHP) and genetically modified mouse models have been used. Considering HIV-1 does not infect NHPs and rodents, recapitulating the human disease requires genetic manipulation of the host or HIV, thereby making it more difficult to translate these animal studies to the in vivo scenario [28,29,30,31,32,33].

Recent advancements in stem cell technologies have enabled researchers to functionally model a diverse range of human organs. Cerebral organoids are self-organized, three-dimensional (3D) cell aggregates that mimic the brain’s cytoarchitecture and the molecular composition of the developing human brain [34,35,36]. Cerebral organoids have been used to model several neurodevelopmental disorders [37,38] and neurotropic infectious diseases such as Zika (ZIKV), human cytomegalovirus (CMV), herpes simplex virus (HSV), and, more recently, SARS-CoV-2 [39,40].

However, as cerebral organoids are neuroectoderm-derived, a major limitation has been the lack of microglia due to their distinct developmental origin from the mesoderm lineage. Several researchers attempted to rectify this deficiency by introducing microglia into cerebral organoids [41,42,43,44]. Alternatively, we have successfully generated cerebral organoids in which microglia grow intrinsically [45]. Given the critical role of the CNS in latency persistence and the limitations of current in vitro culture models, cerebral organoids are quickly gaining interest in the HIV research community. Very recently, Dos reis et al. [44] presented an HIV-infected cerebral organoid model that supports productive viral infection by introducing HIV-infected human primary microglia and immortalized HMC3 microglial cell lines to their 3D cerebral organoid model. In this study, we characterized HIV infection in a cerebral organoid model in which microglia developed intrinsically [45] and related this to the infection of organoid-derived microglia (oMG) and primary human microglia (pMG).

## 2. Materials and Methods

### 2.1. Generation of 3D Human Microglia-Containing Cerebral Organoids

Induced pluripotent stem-cell (iPSC) lines (OH1.5, OH2.6 and OH3.1) were generated from human fibroblasts isolated from skin biopsy samples obtained from 3 healthy donors and have been described before [45,46]. The generation and characterization of the iPSC cell lines were performed by the MIND facility of the UMC. Three-dimensional cerebral organoids were differentiated from the iPSC cell lines, as described before [45]. After matrigel embedment, organoids were transferred to a petri dish and kept in organoid differentiation medium without retinoic acid (RA). Four days later, the medium was supplemented with retinoic acid, and the petri dishes were placed on a belly dancer shaker (stand 4; IBI Scientific BDRLS0001). No changes were made in the composition of the neural induction or organoid differentiation media. Organoids were qualitatively selected for downstream experiments following the previously described guidelines [47].

### 2.2. Generation of 2D Human Cerebral Organoid Dissociates

To assess the susceptibility of cells within the organoids to HIV infection, we first dissociated the 3D organoids to 2D organoid dissociates according to the protocol published by Janssens et al. [48] with some minor modifications. In short, organoids were dissociated using accutase (Innovative Cell Technologies, AT104, San Diego, CA, USA) and plated on matrigel-coated 6-well plates. Organoids were either dissociated separately (single method) or together in a pool of 2–3 organoids (pooled method). After centrifugation, the pellet was collected in organoid differentiation medium with RA and 1 mL organoid suspension was plated per well. After plating, 2D cultures were maintained for 6 days, and 50% of the medium was changed every 2–3 days.

### 2.3. Isolation and Culture of Primary and Organoid-Derived Microglia

Fresh postmortem adult human brain tissue was provided by the Netherlands Brain Bank (NBB). All subjects gave their informed consent for inclusion before they participated in the study. Primary microglia were isolated according to the protocol described before with some minor modifications for human brain tissue [49]. Organoids were dissociated into a single-cell suspension by enzymatic dissociation using papain (18.6 U/mL, Worthington, LK003176, Columbus, OH, USA) and DNAse 1 (337 U/mL, Worthington, LK003170) according to the protocol published before [45]. 

Microglia enrichment was achieved by positive selection for CD11b expression, using magnetic-activated cell sorting (Miltenyi Biotec, Bergisch Gladbach, Germany) according to the manufacturer’s protocol. Primary microglia (pMG) or organoid-derived microglia (oMG) were cultured in poly-L-lysine hydrobromide (PLL)-coated 96-well plates (1 × 10^5^ cells/well) in microglia medium (RPMI 1640; Gibco Life Technologies, Carlsbad, CA, USA) supplemented with 10% FCS, 1% penicillin-streptomycin (Gibco Life Technologies, USA) and 100 ng/mL IL-34 (Miltenyi Biotec, Germany)).

### 2.4. Viral Preparation and HIV Infection 

The HxB2(Balgp160) luciferase reporter viruses (HxB2Luc and HxB2Balgp160Luc) and the NL4-3Balgp160 GFP reporter virus (NL4-3Balgp160GFP) were generated, as described before [26,50]. Hek-293T cells were transfected with the infectious plasmids (HxB2Luc, HxB2Balgp160Luc, and NL4-3Balgp160GFP) using lipofectamine 2000 reagent (Invitrogen). Supernatant containing replication competent virus was harvested 48 h post-transfection and stored at −80 °C until further use. p24 was determined with ELISA p24 assay (Aalto Bioreagent, Dublin, Ireland).

pMG and oMG were infected with 10 ng (p24 Gag) HIVbal (HXB2Balgp160Luc) or HIVbalGFP (NL4-3Balgp160GFP). The medium was fully replaced the next day and cells were cultured in microglia medium for 13–15 days without medium refreshment. For maraviroc (MVC) treatment, pMG and oMG were treated with 200 nM Maraviroc (MVC) for 1 h before infection. The next day, after medium replacement, cells were cultured in microglia medium with 100 nM MVC for 13–15 days without medium refreshment. To analyze new rounds of infection, 100 nM MVC was added to the culture medium 3 days post-infection. All experiments were carried out in duplicate for each condition.

The 2D organoid dissociates and 3D organoids were infected overnight with 100 ng (p24 Gag) HxB2 (HxB2Luc), HIVbal, or HIVbalGFP. The next day, the medium was fully replaced and culture was continued in organoid differentiation medium with RA. For MVC and Raltegravir (RAL) treatment, 2D organoid dissociates and 3D organoids were treated with 200 nM MVC or 400 nM RAL for 2 h before infection. Following medium replacement, culture was continued in organoid differentiation medium with RA with 100 nM MVC or 200 nM RAL. To maximize viral infection, half of the medium was refreshed only 1× per week. All experiments were carried out in duplicate or triplicate. As a negative control for virus capture and release, we also generated empty Matrigel droplets using the same method as we use for embedding cerebral organoids: 30 uL droplets of matrigel (Corning, 356234) were made on indented parafilm and placed in the incubator (37 °C, 5% CO_2_) for 30 min to solidify. Matrigel droplets were then transferred to a 24-well plate and infected with the same protocol as for the 3D organoids.

### 2.5. Luminescence and Immunofluorescence

For luminescence measurements, supernatant was collected 2–3x per week and measured using the Nano-Glo^®^ Luciferase Assay System (Promega) according to the manufacturer’s protocol. Graphs were generated with GraphPad Prism version 8.3.0 (GraphPad Software) and depict the mean and range. 

For immunostaining, 2D organoid dissociates were washed with PBS and fixed with 4% PFA for 2 h at RT. 3D organoids were washed with PBS and fixed with 4% PFA overnight at 4 °C, followed by 30% sucrose solution incubation for 2 days at 4 °C. The 3D organoids were then embedded in tissue tek (VWR, 25608-930) and sectioned at a thickness of 20 µM using a cryostat (Leica CM3050S). The 2D organoid dissociates and 3D organoids were stained according to the protocol described before [45]. See Supplementary Table S1 for antibodies used in this study. Images were obtained with a Zeiss Axio-Scope A1 or Fluoview FV1000 confocal microscope.

### 2.6. Gene Expression Analysis with Real-Time PCR

RNA isolation was performed with the RNeasy kit (Qiagen, Hilden, The Netherlands), including DNAse treatment according to the manufacturer’s protocol. Downstream gene expression analyses were conducted in duplicate from 2 different donors (pMG) or 3 different iPSC lines (2D organoid dissociates and 3D organoids). cDNA synthesis and qPCR were performed as described before [26]. Primer sequences are listed in Supplementary Table S2. Absolute gene expression levels were determined (2ΔCT) and normalized to the reference gene Beta-actin (ACTB). Graphs were generated using GraphPad Prism version 8.3.0 (GraphPad Software).

### 2.7. Single-Cell Sequencing of Cerebral Organoids with SORT-Seq

To further assess the expression of HIV receptors in cerebral organoids at the single-cell level, we made use of an available cerebral organoid single-cell dataset (Kübler in preparation) focused on the inflammatory responses of cerebral organoids. Cerebral organoids generated from OH1.5 and OH2.6 were dissociated at week 9 of differentiation. Viable single cells were FACS sorted based on 7AAD (Dead/alive) and CD45 expression (microglia) into 384-well plates, called cell capture plates, which were ordered from Single Cell Discoveries, a single-cell sequencing service provider based in the Netherlands. Each well of a cell capture plate contains a small, 50 nl droplet of barcoded primers and 10 µL of mineral oil (Sigma M8410). After sorting, plates were immediately centrifuged, snap-frozen, and shipped on dry ice to Single Cell Discoveries, where single-cell RNA sequencing was performed according to an adapted version of the SORT-seq protocol (Muraro et al. [51] with primers described in van den Brink et al. [52]). Cells were heat-lysed at 65 °C followed by cDNA synthesis. After second-strand cDNA synthesis, all the barcoded material from one plate was pooled into one library and amplified using in vitro transcription (IVT). Following amplification, library preparation was performed following the CEL-Seq2 protocol [53] to prepare a cDNA library for sequencing using TruSeq small RNA primers (Illumina). The DNA library was paired-end sequenced on an Illumina Nextseq™ 500, high output, with a 1 × 75 bp Illumina kit (read 1: 26 cycles, index read: 6 cycles, read 2: 60 cycles).

During sequencing, Read 1 was assigned 26 base pairs and was used to identify the Illumina library barcode, cell barcode, and UMI. Read 2 was assigned 60 base pairs and used to map to the reference transcriptome Homo sapiens hg19 (including mitochondrial genes) with BWA-MEM [54]. Data was demultiplexed, as described in Grün et al. [55]. Mapping and generation of count tables were automated using the MapAndGo script [56]. Unsupervised clustering and differential gene expression analysis was performed with the Seurat R toolkit [57,58].

Briefly, we merged each plate’s count matrix into a single Seurat object and then applied stringent quality control (QC) metrics to filter out cells. Before QC, we removed ERCC spike-in genes and genes that were expressed in less than five cells. Based on the distribution of UMI and gene counts per cell, we removed 950 cells with less than 1.000 UMI counts and 400 gene counts. Raw counts were log-normalized with the NormalizeCounts function. Normalized counts were corrected for variance from log UMI count, cell line, days in vitro, and plate covariates using the ScaleData function. Using the corrected count matrix, we constructed a shared nearest-neighbor graph with the FindNeighbours function using the first 14 principal components. We calculated clusters based on the Leiden algorithm [59] with the FindClusters function (resolution = 0.6, iterations = 20). tSNE plots were calculated with the RunTSNE command. Marker genes for each cluster were identified with the Wilcox rank sum test using the FindAllMarkers function. Clusters were annotated with cell-type identity by calculating gene-set enrichment odds ratio and median log-fold change of cell-type gene sets per cluster. Gene sets were extracted from organoid studies by Kanton et al. and Quadrato et al. [60,61]. We used a microglia core signature list by Patir et al. [62] to identify organoid-derived microglia.

## 3. Results

### 3.1. Cerebral Organoids Contain Microglia, Astrocytes, and Neurons

**Figure 1:**
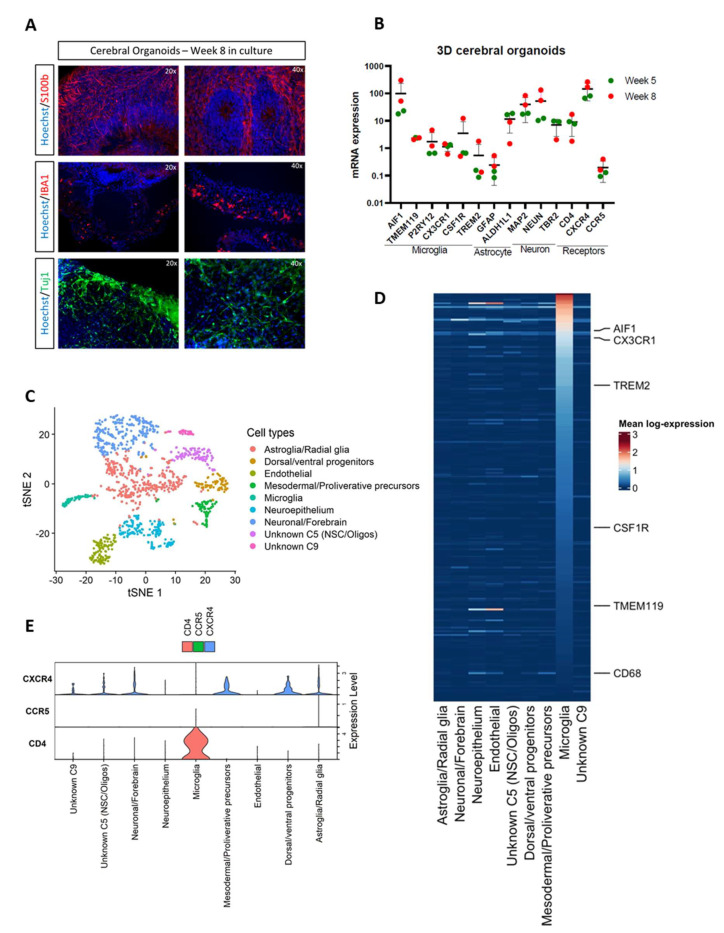
Cerebral organoids exhibit microglia, astrocyte, and neuronal cells and express HIV receptors. (**A**) Double immunostainings of S100b (astrocyte), IBA1 (microglia), and Tuj1 (neurons) combined with nuclear staining Hoechst after 8 weeks in culture. Representative pictures of cerebral organoids from iPSC line OH1.5 are shown. Magnification of 20× and 40× was used. (**B**) mRNA expression levels of microglia, astrocyte, and neuron-specific markers and HIV receptors were assessed by qRT-PCR. Gene expression was normalized to the reference gene ACTB. The means ± standard errors of the means are shown. (**C**–**E**) SORT-seq data of LPS-stimulated organoids after 9-10 weeks in culture. (**C**) Annotated clusters on tSNE plot. Cluster 5 (C5) was only partially annotated. Cluster 9 (C9) was not annotated. (**D**) Heatmap of Patir et al. (2019) microglia signature gene expression across each cluster. (**E**) Violin plots of CD4, CCR5, and CXCR4 gene expression. Expression levels shown are log-normalized and covariate corrected.

Human iPSCs were differentiated into microglia-containing cerebral organoids according to the protocol previously described [45]. These organoids contain intrinsically grown microglia (Iba1), astrocytes (S100b), and neurons (Tuj1) and express specific genes for microglia (*AIF1*, *TMEM119*, *P2RY12*, *CX3CR1*, *CSF1R*, *TREM2*), astrocytes (*GFAP*, *ALDH1L1*), and neurons (*MAP2*, *NEUN*, *TBR2*) (Figure 1A,B). Single-cell RNA sequencing confirmed the presence of cell clusters enriched with markers for microglia, neurons, astroglia, endothelial cells, oligodendrocytes, and a variety of CNS progenitor and precursor cells (Figure 1C). As we have previously shown by bulk RNA-seq analysis on oMG [45], we show that the microglia cluster from the single-cell analysis exhibits a consistently high expression of microglia signature genes [62], including markers that are often not expressed on other microglial culture models, such as *TMEM119* [26] (Figure 1D). Next, we assessed the expression of the main HIV receptors (*CD4*, *CXCR4* and *CCR5*) required for viral entry. *CD4* expression was detected on microglia, whereas *CXCR4* was mostly detected on non-microglia cell clusters, including astroglia, neuronal/forebrain, and dorsal/ventral progenitors, in line with other cerebral organoid scRNA-seq datasets [60,61] (Figure 1E). *CCR5* expression was detected on a small fraction of microglial cells and not on other cell clusters, suggesting that without targeted sequencing approaches, SORT-seq reaches a detection threshold concerning this gene.

### 3.2. Organoid-Derived Microglia Support HIV Infection via the CCR5 Receptor

**Figure 2:**
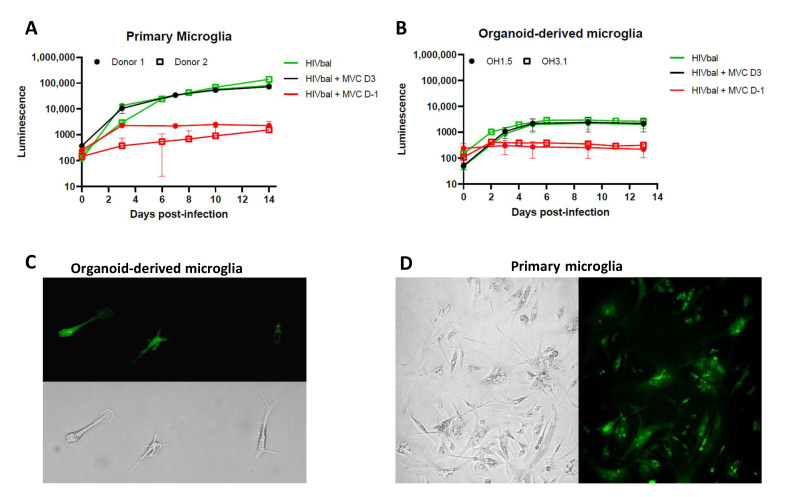
Primary microglia and organoid-derived microglia support productive HIV infection. (**A**) Organoid-derived microglia and (**B**) primary microglia were infected with HIVbal and treated with MVC (200 nM) pre-infection (D-1) and 3 days post-infection (D3). Supernatant was collected at each timepoint and analyzed for luciferase activity. The means ± standard errors of the means are shown. Representative pictures of (**C**) organoid-derived microglia (OH1.5) and (**D**) primary microglia infected with HIVbalGFP, taken in culture after 9 (oMG) and 11 days (pMG) of infection. All pictures were taken with a magnification of 20×.

Microglia grown intrinsically within organoids (oMG) have been extensively characterized and reported to resemble primary microglia at the whole-transcriptome and functional level [45]. To determine whether oMG are susceptible to HIV infection, we isolated microglia from 3D cerebral organoids generated from two iPSC lines (OH1.5 and OH3.1) and infected them with 10 ng (p24 Gag) of CCR5 M-tropic HIV-1 HxB2Balgp160 Luciferase reporter virus (HIVbal). Using the same experimental conditions, viral infections were performed in human primary microglia (pMG). Virus production was measured in the form of luminescence released over time. oMG were found to support HIV infection and production; however, contrary to pMG that show a continuous increase in virus production up to day 14, oMG reached peak infection on day 6 (Figure 2A,B). This may be explained by the higher sensitivity to cell death we observed in oMG compared to primary microglia (Supplementary Figure S1).

The addition of Maraviroc (MVC), a CCR5 inhibitor, before infection (Day-1) restricted viral infection in both oMG and pMG, indicating specific viral entry via the CCR5 coreceptor (Figure 2A,B). Interestingly, the addition of MVC on day 3 post-infection showed no viral suppression, suggesting that the increase in luminescence observed in pMG and oMG is due to continued viral production, as opposed to new rounds of infection after day 3. Furthermore, susceptibility of oMG to HIV infection was confirmed by the detection of GFP+ oMG following infection with 10 ng (p24 Gag) of HIV-1 NL4-3Balgp160 GFP reporter virus (HIVbalGFP) (Figure 2C). The distribution of intracellular GFP protein and fluorescence intensity of GFP+ oMG was similar to GFP+ pMG (Figure 2D).

### 3.3. 2D Organoid Dissociates

**Figure 3:**
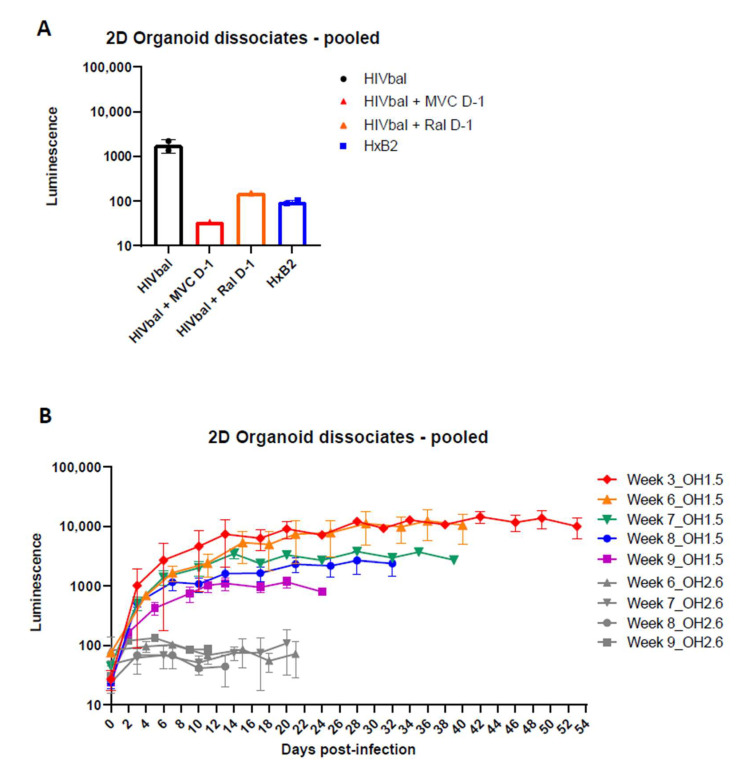
Two-dimensional organoid dissociates support productive HIV-1 infection. (**A**) Week 5 2D organoid dissociates were infected with HIVbal and HxB2 and treated with MVC (100 nM) and Ral (200 nM) pre-infection (D-1). Bar graphs represent luciferase activity measured in supernatant collected on Day 14, post-infection. (**B**) 2D organoid dissociates starting from week 3 up to week 9 were infected with HIVbal. Cerebral organoids were derived from iPSC line OH1.5 or OH2.6. Supernatants were collected at each timepoint. Both graphs depict the means ± standard errors of the means.

**Figure 4:**
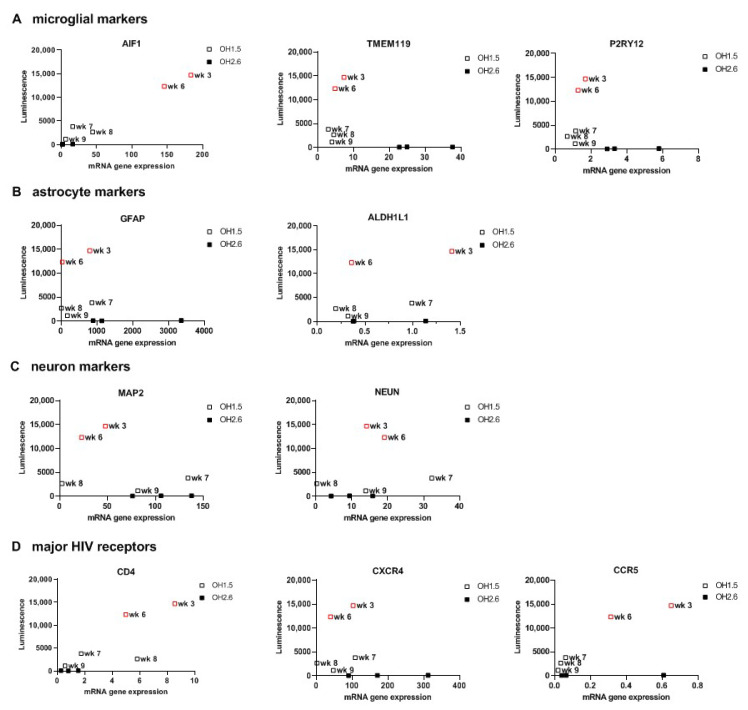
mRNA expression levels of microglia, astrocyte, and neuron markers and HIV receptors in 2D Organoid dissociates. (**A**) Microglia, (**B**) astrocyte, (**C**) neuron markers, and (**D**) HIV receptor expression levels were assessed by qRT-PCR and plotted against the highest luminescence value of corresponding 2D organoid dissociates. Red squares depict the organoid dissociates with the highest luminescence values. Cerebral organoids were derived from iPSC line OH1.5 or OH2.6. Gene expression was normalized to the reference gene ACTB.

After confirming HIV infection of oMG, we investigated whether HIV infection is supported in the microenvironment of the cerebral organoid. First, we enzymatically dissociated 3D organoids to 2D organoid dissociates, which were subsequently kept in culture for 7 days to re-establish cellular interactions in 2D culture [48]. Infection of 2D organoid dissociates with 100 ng (p24 Gag) HIVbal showed viral infection and continuous virus production up to day 14 that could be inhibited by the addition of MVC or Raltegravir (Ral) before infection (Day-1) (Figure 3A). Infection of 2D organoid dissociates with the CXCR4 tropic HIV-1 HxB2-luciferase reporter virus (HxB2) was not supported. Ormel et al., 2018 [45], reported higher numbers of ramified microglia and the expression of mature microglial markers in 3D organoids cultured for 5 weeks compared to 3 weeks; therefore, we evaluated whether the susceptibility to HIV infection is influenced by the developmental stage of the organoid by generating a timeline starting from week 3 and up to week 9 in culture. Interestingly, we observed higher viral production in week 3 and 6 compared to week 7, 8, and 9 (Figure 3B). Gene expression analysis revealed a higher expression of *AIF1*, *CD4*, and *CCR5* in week 3 and week 6 organoid dissociates, whereas the expression of astrocyte- and neuron-specific markers was generally similar to the organoid dissociates of week 7, 8, and 9 (Figure 4). This suggests that the susceptibility of 2D organoid dissociates to HIV infection is dependent on the prevalence of microglia and the expression of *CD4* and *CCR5*. Accordingly, 2D organoid dissociates generated from the OH2.6 iPSC line were not susceptible to HIV infection, most likely due to the low expression of *CD4* and *CCR5*, despite having a high expression of microglia-specific markers (Figure 3B and Figure 4). 

**Figure 5:**
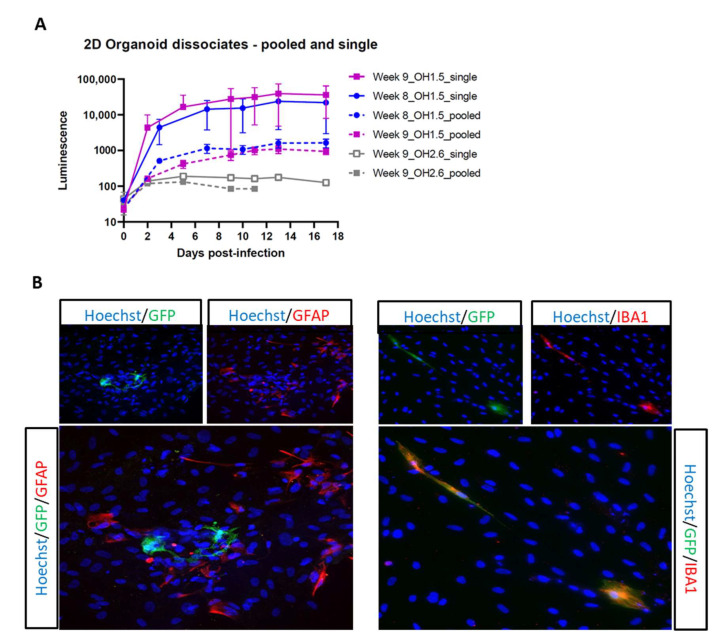
Optimization of 2D organoid dissociates HIV infection. (**A**) 2D organoid dissociates (OH1.5 and OH2.6), dissociated via the pooled and single method, were infected with HIVbal. Supernatant was collected at each timepoint and analyzed for luciferase activity. The means ± standard errors of the means are shown. (**B**) Double immunostainings of GFP combined with GFAP (astrocyte) and IBA1 (microglia) at day 7 post-infection of OH1.5 2D organoid dissociates with HIVbalGFP. All pictures were taken with a magnification of 20×.

Next, we sought to enhance viral infection in these 2D organoid dissociates through minor modification of the dissociation protocol, originally described by Janssens et al., 2019 [48]. Briefly, we dissociated each organoid separately, instead of a pool of three organoids, then plated the cell suspension of each organoid in a Matrigel-coated 6-well plate so that each well contained the cell suspension derived from one organoid. With this new dissociation protocol, referred to as single dissociation, we were able to increase viral infection by 10-fold (Figure 5A). Despite the optimized protocol, 2D organoid dissociates from the OH2.6 iPSC line did not support HIV infection, highlighting the importance of choosing the right iPSC line for generating cerebral organoids that are susceptible to HIV infection. To further confirm HIV infection of OH1.5-derived 2D organoid dissociates, and to determine the target cells of HIV, we infected 2D organoid dissociates with HIVbalGFP. GFP was detected exclusively in microglia (Iba1+) within 4 days of infection, indicating that, within the 2D cerebral model system, microglia are the only HIV target cells (Figure 5B).

### 3.4. 3D Cerebral Organoids

**Figure 6:**
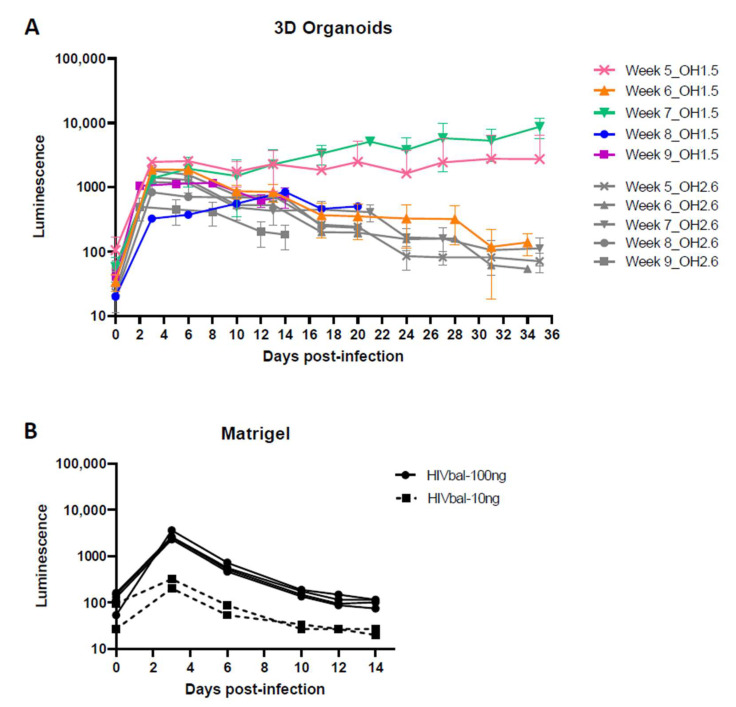
HIV infection of 3D organoids. (**A**) Three-dimensional cerebral organoids, starting from week 5 up to week 9, were infected with 100 ng (p24 Gag) HIVbal. Cerebral organoids were derived from iPSC line OH1.5 and OH2.6. (**B**) Matrigel droplets were infected with 10 ng and 100 ng (p24 Gag) HIVbal. Supernatant was collected at each timepoint and analyzed for luciferase activity. The means ± standard errors of the means are shown.

Following the successful infection of the 2D organoid dissociates, we proceeded to infect 3D cerebral organoids with HIVbal, starting at week 5 up to week 9. Contrary to the 2D organoid dissociates that showed continuous virus production, peak infection was reached within the first week of infection and steadily decreased in the following weeks, except for week 5 and week 7 organoids (Figure 6A). A similar infectivity and infection pattern was unexpectedly also observed in the OH2.6 iPSC line, despite not being susceptible to HIV infection in the 2D organoid dissociates. 

**Figure 7:**
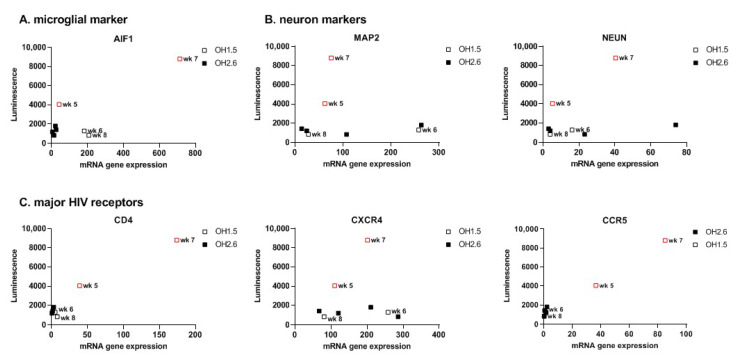
mRNA expression levels of microglia and neuron markers and HIV receptors in 3D Organoids. (**A**) Microglia and (**B**) neuron markers and (**C**) HIV receptor expression levels were assessed by qRT-PCR and plotted against the highest luminescence value of corresponding 3D organoid. Red squares depict 3D organoids with continued luminescence release. Cerebral organoids were derived from iPSC line OH1.5 and OH2.6. Gene expression was normalized to the reference gene ACTB.

To get a better understanding of the luminescence observed from these OH2.6-derived organoids, we investigated whether Matrigel, used for organoid embedment, has any effect on the release of the luciferase protein into the culture medium. Infection of Matrigel droplets with HIVbal resulted in the release of luminescence over time, which was consistent with the luminescence pattern observed after infection of 3D organoids (Figure 6B). This suggests that, except for week 5 and week 7 organoids, the luminescence measured was most likely due to luciferase diffusion from the Matrigel and not a result of productive viral infection. Accordingly, *CD4* and *CCR5* gene expression was exclusively found in week 5 and week 7 organoids (Figure 7). Week 7 organoids also showed high expression of *AIF1*. Taken together, this highlights that caution should be taken when using the release of luciferase in the culture medium as a measurement for viral production.

**Figure 8:**
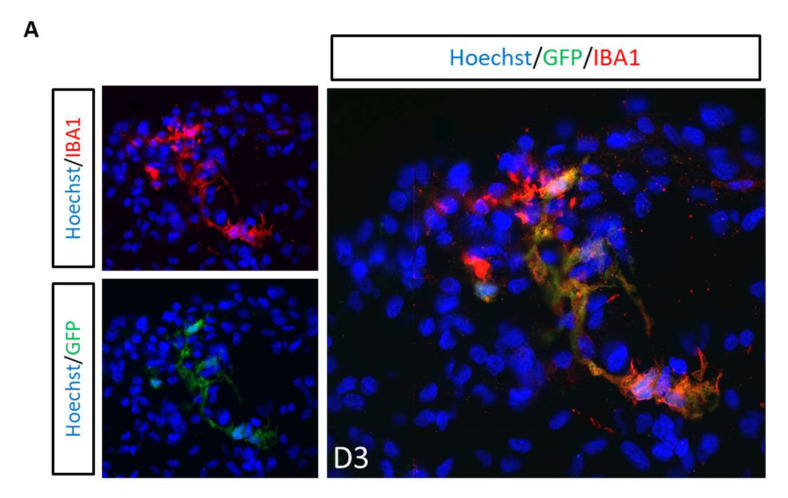

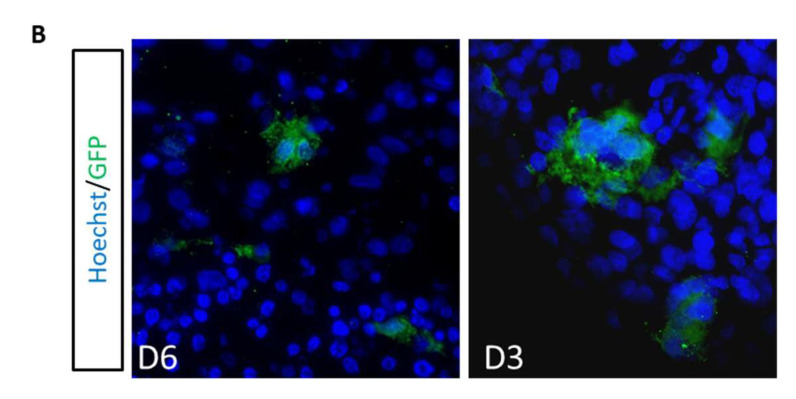
Microglia are the only HIV target cells. (**A**) Double immunostainings of 3D cerebral organoids (OH1.5) with GFP combined with IBA1 (microglia) at day 3 post-infection with NL4-3Balgp160GFP. (**B**) Representative pictures of GFP+ multinucleated microglia (IBA1+) in 3D cerebral organoids at day 3 and day 6 post-infection with HIVbalGFP. All pictures were taken with a magnification of 40×.

Lastly, we investigated whether the microglia grown within the organoids can mimic the multinucleated pathology of HIV-infected microglia often observed in human postmortem brain tissue. Three-dimensional cerebral organoids were infected with HIVbalGFP. GFP+ cells were found exclusively in microglia (Iba1+) within 3 days of infection, further confirming that microglia are the only target cells of HIV in the 3D organoid model system (Figure 8A). Interestingly, GFP+ cells also had the characteristic multinucleation observed in postmortem human brain tissue (Figure 8B).

## 4. Discussion

To advance our knowledge on the CNS viral reservoir, having a good human representative CNS culture model is essential to study the underlying mechanisms of HIV-1 CNS infection, persistence, and reversal. Cerebral organoids are proposed to become a powerful tool to model the human CNS and advance NeuroHIV Research, with multiple application possibilities [63]. In this study, we characterized microglia-containing human cerebral organoids in the context of HIV infection. We demonstrated productive HIV infection in organoid-derived microglia, 2D organoid dissociates, and 3D organoids. HIV infection could be successfully inhibited with MVC, indicating that infection is mediated through the CD4 and CCR5 co-receptors. Interestingly, the addition of MVC on day 3 revealed that infectivity takes place mainly within the first 3 days of infection, followed by continued virus production up to day 6 in oMG and day 14 in pMG. This finding, however, is inconsistent with fluorescence images showing an increase in GFP+ cells in culture over time, suggesting new rounds of infection [9] (Supplementary Figure S2). However, contrary to the continuous virus production and viral spread we observed in cultured pMG, HIV infection in postmortem brain tissue is reported to be focally distributed in about 1 to 10% of CD68+ microglia/macrophage cells, irrespective of ART treatment and/or HAND [10,11,12,13,14]. This sporadic detection of viral genome was also observed in SIV-infected macaque models, with an infectivity of 0.268 and 231 IUPM (infectious units per million) in treated and untreated macaques [64,65,66,67,68]. These findings suggest that HIV infection in the brain is limited and takes place without viral spread. In this regard, the low infection of HIV in oMG, 2D organoid dissociates, and 3D organoids, as compared to cultured pMG, is reflective of the limited HIV-infected microglial population observed in vivo and in non-human primates. Interestingly, within the organoids, we also observed microglia clusters in which all cells were infected as well as uninfected Iba1+ cells that were in close proximity to infected cells, suggesting a limitation in viral spread (Supplementary Figures S3 and S4). Nonetheless, it remains to be addressed whether the decrease in viral production observed in oMG after the first week of infection is due to a higher cell death rate or, potentially, an increased susceptibility to HIV latency compared to pMG. A small number of astrocytes (0.4–5.2%) have been reported to be infected by HIV-1, both in vivo and in vitro, mainly through endocytosis [10,12,15,16,19,69,70,71,72]. Within our organoids, HIV infection was only observed in microglia. This is consistent with dos Reis et al., 2020 [44] and Ryan et al., 2020 [27], in which microglia were found to be the only HIV target cells within their human brain organoid (hBORG) and hiPSC-based tri-culture model of neurons, astrocytes and microglia. However, we acknowledge that our experiments were performed on organoids in which astrocytes have not yet fully matured and should be repeated with older organoids with fully matured astrocytes [73]. Furthermore, although the CXCR4 receptor is expressed on neurons and, to a lesser extent, astrocytes, cerebral organoids were not susceptible to the CXCR4-using HxB2 lab strain, most likely due to the lack of co-expression with the CD4 receptor. 

In line with this finding, susceptibility to HIV infection with HIVbal was found to be highly dependent on the co-expression of the CD4 and CCR5 receptor genes and the microglia-specific marker AIF1 rather than the maturation of the organoid, suggesting that intrinsically grown microglia are susceptible to HIV infection, irrespective of maturation. This finding, however, highlights an important limitation of the organoid model, which is the variability between organoids from the same batch and across iPSC lines, most likely caused by the self-patterning-based development. Consequently, although OH1.5- and OH2.6-derived organoids were generated with the same protocol and under the same conditions, OH2.6-derived organoids had very limited expression of the HIV receptors and were not susceptible to HIV infection despite the expression of microglia-specific markers (TMEM119 and P2RY12). Several groups have been able to minimize heterogeneity by substituting Matrigel for polymer-based scaffolds [74], removing Matrigel before spinning culture [75], or the use of miniaturized spinning bioreactors for organoid culture [76,77]. Alternatively, cerebral organoids can also be generated in the presence of exogenous patterning factors to generate specific brain regions with less heterogeneity than unguided protocols [78]. Nonetheless, we highly recommend researchers perform a pilot experiment with cerebral organoids derived from different iPSC lines to determine which iPSC line produces organoids susceptible to HIV infection based on the co-expression of microglia-specific markers and the major HIV receptors, CD4, CXCR4, and CCR5. 

A well-known difficulty of the cerebral organoid model is the development and the ensuing limited number of microglia (~1%) within the organoids. As microglia are the main target cell for HIV in the CNS, having a suitable amount of microglia within the organoids is essential. To mitigate this, Xu et al. [79] co-cultured hPSC-derived primitive macrophage progenitors (PMPs) and primitive neural progenitor cells (NPCs) at the onset of 3D organoid formation to generate microglia-containing brain organoids. By controlling the starting number of the PMPs and NPCs, they were able to control the ratio of microglia within the organoids. Alternatively, iPSC-derived microglia [41,42,43], primary microglia, and the microglial cell line HMC3 [44] have been incorporated into mature cerebral organoids to overcome the lack of microglia differentiation within cerebral organoids during development [26]. 

In addition to the aforementioned obstacles, we caution researchers of practical limitations when using luminescence as a readout for the viral infection of Matrigel-embedded organoids. We observed that Matrigel withholds viral particles and/or luciferase protein despite generous washing post-infection to remove unbound virus and remnants. Therefore, we highly recommend the use of additional methods, such as p24 ELISA, HIV RNA transcripts, and/or a fluorescently labeled viral vector to validate HIV infection. As more protocols are replacing Matrigel, we believe this will also facilitate the use of luciferase-tagged reporter viruses in the future, although it remains important to also assess the effects of these polymer scaffolds when using a luminescence-based readout system. 

The first study to demonstrate the utility of cerebral organoids within HIV research was recently reported by Dos reis and colleagues. In this study, they incorporated HIV-infected HMC3 microglial cell line or human primary microglia into a 3D human brain organoid (hBORG) model that supported virus production and exhibited an increased inflammatory response (TNF-α and IL-1β) [44]. Microglial cell lines have been reported to have large transcriptomic and phenotypic discrepancies with primary microglia, a high proliferation rate, and poor-to-no expression of microglia specific markers (i.e., CX3CR1, P2RY12, and TMEM119) and the major HIV receptors (CD4, CXCR4 and CCR5) [25,26,80]. Therefore, microglial cell lines are limited in their use for HIV research studies. Furthermore, while infection of microglia before incorporation into the cerebral organoids greatly facilitates infectivity, this does not represent the in vivo scenario, as HIV infection of microglia takes place within the CNS in the presence of other CNS cells. Our study, on the other hand, is the first to characterize HIV infection within microglia-containing cerebral organoids. Although a more indepth investigation of the key HIV neuropathological features of HAND (such as neuroinflammation) and neurological damage was not in the scope of this study, we observed several multinucleated GFP+ cells within the organoids that resembled the multinucleation observed in HIV-infected iPSC-derived microglia [27] and cultured primary human microglia [9,81,82] and postmortem brain tissue of HIV-infected individuals.

Altogether, despite the current obstacles of 3D cerebral organoids, the model presented in this paper accounts for several of the shortcomings of 2D monoculture models, postmortem brain tissue biopsies, and animal models currently used for HIV CNS research. Furthermore, with ongoing advancements in cerebral organoid generation and culture to mitigate these limitations, cerebral organoids will become a valuable human-representative 3D CNS culture model to advance neuroHIV research. The use of cerebral organoids within the HIV research field will require the controlled induction of the mesoderm lineage to allow microglia differentiation or incorporation of microglia within organoids to ensure the support of HIV-1 infection. 

To the best of our knowledge, we are the first to report the productive HIV-1 infection of microglia-containing cerebral organoids. This model system can be used to study the impact of HIV infection on the CNS, gain a better understanding of the neuropathogenesis of HAND and HIV latency in the brain, and facilitate the testing of new therapeutic and curative strategies. 

## Data Availability

The datasets generated and analyzed during the current study are available from the corresponding author on reasonable request.

## Supplementary Materials

The following supporting information can be downloaded at: https://www.mdpi.com/article/10.3390/v14040829/s1, Figure S1: Viability of Organoid-derived microglia and primary microglia after HIV infection; Figure S2: Graphical representation of GFP+ distribution in primary microglia infected with HIVbalGFP. Images were taken in culture after 11 days of infection. Magnification of 10× was used; Figure S3: Graphical representation of GFP+ microglial cells within 3D cerebral organoids. (A). Double immunostainings of a 3D cerebral organoid (OH1.5) with GFP combined with IBA1 (microglia) at day 14 post-infection with HIVbalGFP. Magnification of 10× was used. (B). A zoomed-in image of Figure 3A taken at 40× magnification depicting GFP+ /IBA1+ microglia cells; Figure S4: HIV spread within 3D cerebral organoids is limited. Double immunostainings of 3D cerebral organoids (OH1.5) with GFP combined with IBA1 (microglia) at day 14 post-infection with HIVbalGFP. Magnification of 20× was used; Table S1: Antibodies used in this study for immunofluorescence; Table S2: Primer sequences used for qRT-PCR experiments.

## References

[1] Valcour V., Sithinamsuwan P., Letendre S., Ances B. (2011). Pathogenesis of HIV in the central nervous system. Curr. HIV/AIDS Rep..

[2] Spudich S., González-Scarano F. (2012). HIV-1-related central nervous system disease: Current issues in pathogenesis, diagnosis, and treatment. Cold Spring Harb. Perspect. Med..

[3] León-Rivera R., Veenstra M., Donoso M., Tell E., Berman J.W., Eugenin E.A., Morgello S. (2021). Central Nervous System (CNS) Viral Seeding by Mature Monocytes and Potential Therapies To Reduce CNS Viral Reservoirs in the cART Era. MBio.

[4] Heaton R.K., Clifford D.B., Franklin D.R., Woods S.P., Ake C., Vaida F., Ellis R.J., Letendre S.L., Marcotte T.D., Atkinson J.H. (2010). HIV-associated neurocognitive disorders persist in the era of potent antiretroviral therapy: Charter Study. Neurology.

[5] Simioni S., Cavassini M., Annoni J.M., Rimbault Abraham A., Bourquin I., Schiffer V., Calmy A., Chave J.P., Giacobini E., Hirschel B. (2010). Cognitive dysfunction in HIV patients despite long-standing suppression of viremia. AIDS.

[6] Clifford D.B., Ances B.M. (2013). HIV-associated neurocognitive disorder. Lancet Infect. Dis..

[7] Jadhav S., Nema V. (2021). HIV-Associated Neurotoxicity: The Interplay of Host and Viral Proteins. Mediators Inflamm..

[8] Borrajo A., Spuch C., Penedo M.A., Olivares J.M., Agís-Balboa R.C. (2021). Important role of microglia in HIV-1 associated neurocognitive disorders and the molecular pathways implicated in its pathogenesis. Ann. Med..

[9] Cenker J.J., Stultz R.D., McDonald D. (2017). Brain Microglial Cells Are Highly Susceptible to HIV-1 Infection and Spread. AIDS Res. Hum. Retroviruses.

[10] Churchill M.J., Gorry P.R., Cowley D., Lal L., Sonza S., Purcell D.F., Thompson K.A., Gabuzda D., McArthur J.C., Pardo C.A. (2006). Use of laser capture microdissection to detect integrated HIV-1 DNA in macrophages and astrocytes from autopsy brain tissues. J. Neurovirol..

[11] Thompson K.A., Cherry C.L., Bell J.E., McLean C.A. (2011). Brain cell reservoirs of latent virus in presymptomatic HIV-infected individuals. Am. J. Pathol..

[12] Trillo-Pazos G., Diamanturos A., Rislove L., Menza T., Chao W., Belem P., Sadiq S., Morgello S., Sharer L., Volsky D.J. (2003). Detection of HIV-1 DNA in microglia/macrophages, astrocytes and neurons isolated from brain tissue with HIV-1 encephalitis by laser capture microdissection. Brain Pathol..

[13] Tso F.Y., Kang G., Kwon E.H., Julius P., Li Q., West J.T., Wood C. (2018). Brain is a potential sanctuary for subtype C HIV-1 irrespective of ART treatment outcome. PLoS ONE.

[14] Ko A., Kang G., Hattler J.B., Galadima H.I., Zhang J., Li Q., Kim W.K. (2019). Macrophages but not Astrocytes Harbor HIV DNA in the Brains of HIV-1-Infected Aviremic Individuals on Suppressive Antiretroviral Therapy. J. Neuroimmune Pharmacol..

[15] Lutgen V., Narasipura S.D., Barbian H.J., Richards M., Wallace J., Razmpour R., Buzhdygan T., Ramirez S.H., Prevedel L., Eugenin E.A. (2020). HIV infects astrocytes in vivo and egresses from the brain to the periphery. PLoS Pathog..

[16] Valdebenito S., Castellano P., Ajasin D., Eugenin E.A. (2021). Astrocytes are HIV reservoirs in the brain: A cell type with poor HIV infectivity and replication but efficient cell-to-cell viral transfer. J. Neurochem..

[17] Li G.-H., Anderson C., Jaeger L., Do T., Major E.O., Nath A. (2015). Cell-to-cell contact facilitates HIV transmission from lymphocytes to astrocytes via CXCR4. Aids.

[18] Russell R.A., Chojnacki J., Jones D.M., Johnson E., Do T., Eggeling C., Padilla-Parra S., Sattentau Q.J. (2017). Astrocytes Resist HIV-1 Fusion but Engulf Infected Macrophage Material. Cell Rep..

[19] Chauhan A., Mehla R., Vijayakumar T.S., Handy I. (2014). Endocytosis-mediated HIV-1 entry and its significance in the elusive behavior of the virus in astrocytes. Virology.

[20] Luo X., He J.J. (2015). Cell–cell contact viral transfer contributes to HIV infection and persistence in astrocytes. J. Neurovirol..

[21] Wallet C., De Rovere M., Van Assche J., Daouad F., De Wit S., Gautier V., Mallon P.W.G., Marcello A., Van Lint C., Rohr O. (2019). Microglial Cells: The Main HIV-1 Reservoir in the Brain. Front. Cell. Infect. Microbiol..

[22] Al-Harthi L., Joseph J., Nath A. (2019). Correction to: Astrocytes as an HIV CNS reservoir: highlights and reflections of an NIMH-sponsored symposium. J. Neurovirol..

[23] Alvarez-Carbonell D., Ye F., Ramanath N., Garcia-Mesa Y., Knapp P.E., Hauser K.F., Karn J. (2019). Cross-talk between microglia and neurons regulates HIV latency. PLoS Pathog..

[24] Brese R.L., Gonzalez-Perez M.P., Koch M., O’Connell O., Luzuriaga K., Somasundaran M., Clapham P.R., Dollar J.J., Nolan D.J., Rose R. (2018). Ultradeep single-molecule real-time sequencing of HIV envelope reveals complete compartmentalization of highly macrophage-tropic R5 proviral variants in brain and CXCR4-using variants in immune and peripheral tissues. J. Neurovirol..

[25] Rai M.A., Hammonds J., Pujato M., Mayhew C., Roskin K., Spearman P. (2020). Comparative analysis of human microglial models for studies of HIV replication and pathogenesis. Retrovirology.

[26] Gumbs S.B.H., Kübler R., Gharu L., Schipper P.J., Borst A.L., Snijders G.J.L.J., Ormel P.R., van Berlekom A.B., Wensing A.M.J., de Witte L.D. (2022). Human microglial models to study HIV infection and neuropathogenesis: a literature overview and comparative analyses. J. Neurovirol..

[27] Ryan S.K., Gonzalez M.V., Garifallou J.P., Bennett F.C., Williams K.S., Sotuyo N.P., Mironets E., Cook K., Hakonarson H., Anderson S.A. (2020). Neuroinflammation and EIF2 Signaling Persist despite Antiretroviral Treatment in an hiPSC Tri-culture Model of HIV Infection. Stem Cell Reports.

[28] Joseph J. (2018). Optimizing animal models for HIV-associated CNS dysfunction and CNS reservoir research. J. Neurovirol..

[29] Beck S.E., Queen S.E., Metcalf Pate K.A., Mangus L.M., Abreu C.M., Gama L., Witwer K.W., Adams R.J., Zink M.C., Clements J.E. (2018). An SIV/macaque model targeted to study HIV-associated neurocognitive disorders. J. Neurovirol..

[30] Moretti S., Virtuoso S., Sernicola L., Farcomeni S., Maggiorella M.T., Borsetti A. (2021). Advances in SIV/SHIV Non-Human Primate Models of NeuroAIDS. Pathogens.

[31] Mallard J., Williams K.C. (2018). Animal models of HIV-associated disease of the central nervous system. Handb. Clin. Neurol..

[32] Honeycutt J.B., Garcia J.V. (2018). Humanized mice: models for evaluating NeuroHIV and cure strategies. J. Neurovirol..

[33] Jaeger L.B., Nath A. (2012). Modeling HIV-associated neurocognitive disorders in mice: New approaches in the changing face of HIV neuropathogenesis. DMM Dis. Model. Mech..

[34] Camp J.G., Badsha F., Florio M., Kanton S., Gerber T., Wilsch-Bräuninger M., Lewitus E., Sykes A., Hevers W., Lancaster M. (2015). Human cerebral organoids recapitulate gene expression programs of fetal neocortex development. Proc. Natl. Acad. Sci. USA.

[35] Pasca A.M., Sloan S.A., Clarke L.E., Tian Y., Makinson C.D., Huber N., Kim C.H., Park J.Y., O’Rourke N.A., Nguyen K.D. (2015). Functional cortical neurons and astrocytes from human pluripotent stem cells in 3D culture. Nat. Methods.

[36] Lancaster M.A., Renner M., Martin C.A., Wenzel D., Bicknell L.S., Hurles M.E., Homfray T., Penninger J.M., Jackson A.P., Knoblich J.A. (2013). Cerebral organoids model human brain development and microcephaly. Nature.

[37] Adams J.W., Cugola F.R., Muotri A.R. (2019). Brain Organoids as Tools for Modeling Human Neurodevelopmental Disorders. Physiology.

[38] Sun N., Meng X., Liu Y., Song D., Jiang C., Cai J. (2021). Applications of brain organoids in neurodevelopment and neurological diseases. J. Biomed. Sci..

[39] Agboola O.S., Hu X., Shan Z., Wu Y., Lei L. (2021). Brain organoid: a 3D technology for investigating cellular composition and interactions in human neurological development and disease models in vitro. Stem Cell Res. Ther..

[40] Fan W., Christian K.M., Song H., Ming G. (2022). Applications of Brain Organoids for Infectious Diseases. J. Mol. Biol..

[41] Abud E.M., Ramirez R.N., Martinez E.S., Healy L.M., Nguyen C.H.H., Newman S.A., Yeromin A.V., Scarfone V.M., Marsh S.E., Fimbres C. (2017). iPSC-Derived Human Microglia-like Cells to Study Neurological Diseases. Neuron.

[42] Abreu C.M., Gama L., Krasemann S., Chesnut M., Odwin-Dacosta S., Hogberg H.T., Hartung T., Pamies D. (2018). Microglia Increase Inflammatory Responses in iPSC-Derived Human BrainSpheres. Front. Microbiol..

[43] Song L., Yuan X., Jones Z., Vied C., Miao Y., Marzano M., Hua T., Sang Q.-X.A., Guan J., Ma T. (2019). Functionalization of Brain Region-specific Spheroids with Isogenic Microglia-like Cells. Sci. Rep..

[44] dos Reis R.S., Sant S., Keeney H., Wagner M.C.E., Ayyavoo V. (2020). Modeling HIV-1 neuropathogenesis using three-dimensional human brain organoids (hBORGs) with HIV-1 infected microglia. Sci. Rep..

[45] Ormel P.R., Vieira de Sá R., van Bodegraven E.J., Karst H., Harschnitz O., Sneeboer M.A.M., Johansen L.E., van Dijk R.E., Scheefhals N., Berdenis van Berlekom A. (2018). Microglia innately develop within cerebral organoids. Nat. Commun..

[46] Harschnitz O., van den Berg L.H., Johansen L.E., Jansen M.D., Kling S., Vieira de Sá R., Vlam L., van Rheenen W., Karst H., Wierenga C.J. (2016). Autoantibody pathogenicity in a multifocal motor neuropathy induced pluripotent stem cell-derived model. Ann. Neurol..

[47] Lancaster M.A., Knoblich J.A. (2014). Generation of cerebral organoids from human pluripotent stem cells. Nat. Protoc..

[48] Janssens S., Schotsaert M., Manganaro L., Dejosez M., Simon V., García-Sastre A., Zwaka T.P. (2019). FACS-Mediated Isolation of Neuronal Cell Populations From Virus-Infected Human Embryonic Stem Cell-Derived Cerebral Organoid Cultures. Curr. Protoc. Stem Cell Biol..

[49] Mattei D., Ivanov A., van Oostrum M., Pantelyushin S., Richetto J., Mueller F., Beffinger M., Schellhammer L., Vom Berg J., Wollscheid B. (2020). Enzymatic dissociation induces transcriptional and proteotype bias in brain cell populations. Int. J. Mol. Sci..

[50] Lebbink R.J., De Jong D.C.M., Wolters F., Kruse E.M., Van Ham P.M., Wiertz E.J.H.J., Nijhuis M. (2017). A combinational CRISPR/Cas9 gene-editing approach can halt HIV replication and prevent viral escape. Sci. Rep..

[51] Muraro M.J., Dharmadhikari G., Grün D., Groen N., Dielen T., Jansen E., van Gurp L., Engelse M.A., Carlotti F., de Koning E.J.P. (2016). A Single-Cell Transcriptome Atlas of the Human Pancreas. Cell Syst..

[52] Van Den Brink S.C., Sage F., Vértesy Á., Spanjaard B., Peterson-Maduro J., Baron C.S., Robin C., Van Oudenaarden A. (2017). Single-cell sequencing reveals dissociation-induced gene expression in tissue subpopulations. Nat. Methods.

[53] Hashimshony T., Senderovich N., Avital G., Klochendler A., de Leeuw Y., Anavy L., Gennert D., Li S., Livak K.J., Rozenblatt-Rosen O. (2016). CEL-Seq2: sensitive highly-multiplexed single-cell RNA-Seq. Genome Biol..

[54] Anders S., Huber W. (2010). Differential expression analysis for sequence count data. Genome Biol..

[55] Grün D., Kester L., Van Oudenaarden A. (2014). Validation of noise models for single-cell transcriptomics. Nat. Methods.

[56] anna-alemany/transcriptomics · GitHub. https://github.com/anna-alemany/transcriptomics/tree/master/mapandgo.

[57] Butler A., Hoffman P., Smibert P., Papalexi E., Satija R. (2018). Integrating single-cell transcriptomic data across different conditions, technologies, and species. Nat. Biotechnol..

[58] Hao Y., Hao S., Andersen-Nissen E., Mauck W.M., Zheng S., Butler A., Lee M.J., Wilk A.J., Darby C., Zager M. (2021). Integrated analysis of multimodal single-cell data. Cell.

[59] Traag V.A., Waltman L., van Eck N.J. (2019). From Louvain to Leiden: guaranteeing well-connected communities. Sci. Rep..

[60] Kanton S., Boyle M.J., He Z., Santel M., Weigert A., Sanchís-Calleja F., Guijarro P., Sidow L., Fleck J.S., Han D. (2019). Organoid single-cell genomic atlas uncovers human-specific features of brain development. Nature.

[61] Quadrato G., Nguyen T., Macosko E.Z., Sherwood J.L., Yang S.M., Berger D.R., Maria N., Scholvin J., Goldman M., Kinney J.P. (2017). Cell diversity and network dynamics in photosensitive human brain organoids. Nature.

[62] Patir A., Shih B., McColl B.W., Freeman T.C. (2019). A core transcriptional signature of human microglia: Derivation and utility in describing region-dependent alterations associated with Alzheimer’s disease. Glia.

[63] Premeaux T.A., Mediouni S., Leda A., Furler R.L., Valente S.T., Fine H.A., Nixon D.F., Ndhlovu L.C. (2021). Next-Generation Human Cerebral Organoids as Powerful Tools To Advance NeuroHIV Research. MBio.

[64] Avalos C.R., Price S.L., Forsyth E.R., Pin J.N., Shirk E.N., Bullock B.T., Queen S.E., Li M., Gellerup D., O’Connor S.L. (2016). Quantitation of Productively Infected Monocytes and Macrophages of Simian Immunodeficiency Virus-Infected Macaques. J. Virol..

[65] Avalos C.R., Abreu C.M., Queen S.E., Li M., Price S., Shirk E.N., Engle E.L., Forsyth E., Bullock B.T., Mac Gabhann F. (2017). Brain macrophages in simian immunodeficiency virus-infected, antiretroviral-suppressed macaques: A functional latent reservoir. MBio.

[66] Abreu C., Shirk E.N., Queen S.E., Beck S.E., Mangus L.M., Pate K.A.M., Mankowski J.L., Gama L., Clements J.E. (2019). Brain macrophages harbor latent, infectious simian immunodeficiency virus. AIDS.

[67] Abreu C.M., Veenhuis R.T., Avalos C.R., Graham S., Parrilla D.R., Ferreira E.A., Queen S.E., Shirk E.N., Bullock B.T., Li M. (2019). Myeloid and CD4 T Cells Comprise the Latent Reservoir in Antiretroviral Therapy-Suppressed SIVmac251-Infected Macaques. MBio.

[68] Gama L., Abreu C., Shirk E.N., Queen S.E., Beck S.E., Metcalf Pate K.A., Bullock B.T., Zink M.C., Mankowski J.L., Clements J.E. (2018). SIV Latency in Macrophages in the CNS. Curr. Top. Microbiol. Immunol..

[69] Liu Y., Liu H., Kim B.O., Gattone V.H., Li J., Nath A., Blum J., He J.J. (2004). CD4-independent infection of astrocytes by human immunodeficiency virus type 1: requirement for the human mannose receptor. J. Virol..

[70] Churchill M.J., Wesselingh S.L., Cowley D., Pardo C.A., McArthur J.C., Brew B.J., Gorry P.R. (2009). Extensive astrocyte infection is prominent in human immunodeficiency virus-associated dementia. Ann. Neurol..

[71] Gray L.R., Turville S.G., Hitchen T.L., Cheng W.J., Ellett A.M., Salimi H., Roche M.J., Wesselingh S.L., Gorry P.R., Churchill M.J. (2014). HIV-1 Entry and Trans-Infection of Astrocytes Involves CD81 Vesicles. PLoS ONE.

[72] Li G.-H., Maric D., Major E.O., Nath A. (2020). Productive HIV infection in astrocytes can be established via a nonclassical mechanism. AIDS.

[73] Sloan S.A., Darmanis S., Huber N., Khan T.A., Birey F., Caneda C., Reimer R., Quake S.R., Barres B.A., Paşca S.P. (2017). Human Astrocyte Maturation Captured in 3D Cerebral Cortical Spheroids Derived from Pluripotent Stem Cells. Neuron.

[74] Lancaster M.A., Corsini N.S., Wolfinger S., Gustafson E.H., Phillips A.W., Burkard T.R., Otani T., Livesey F.J., Knoblich J.A. (2017). Guided self-organization and cortical plate formation in human brain organoids. Nat. Biotechnol..

[75] Sivitilli A.A., Gosio J.T., Ghoshal B., Evstratova A., Trcka D., Ghiasi P., Hernandez J.J., Beaulieu J.M., Wrana J.L., Attisano L. (2020). Robust production of uniform human cerebral organoids from pluripotent stem cells. Life Sci. Alliance.

[76] Qian X., Jacob F., Song M.M., Nguyen H.N., Song H., Ming G.L. (2018). Generation of human brain region–specific organoids using a miniaturized spinning bioreactor. Nat. Protoc..

[77] Phelan M.A., Lelkes P.I., Swaroop A. (2018). Mini and customized low-cost bioreactors for optimized high-throughput generation of tissue organoids. Stem Cell Investig..

[78] Xiang Y., Cakir B., Park I.H. (2021). Deconstructing and reconstructing the human brain with regionally specified brain organoids. Semin. Cell Dev. Biol..

[79] Xu R., Boreland A.J., Li X., Erickson C., Jin M., Atkins C., Pang Z.P., Daniels B.P., Jiang P. (2021). Developing human pluripotent stem cell-based cerebral organoids with a controllable microglia ratio for modeling brain development and pathology. Stem Cell Reports.

[80] Melief J., Sneeboer M.A.M., Litjens M., Ormel P.R., Palmen S.J.M.C., Huitinga I., Kahn R.S., Hol E.M., de Witte L.D. (2016). Characterizing primary human microglia: A comparative study with myeloid subsets and culture models. Glia.

[81] Lee S.C., Hatch W.C., Liu W., Brosnan C.F., Dickson D.W. (1993). Productive Infection of Human Fetal Microglia in Vitro by HIV-1. Ann. N. Y. Acad. Sci..

[82] Castellano P., Prevedel L., Eugenin E.A. (2017). HIV-infected macrophages and microglia that survive acute infection become viral reservoirs by a mechanism involving Bim. Sci. Rep..

