# Supplementary material for: Characterization of HIV-1 Infection in Microglia-Containing Human Cerebral Organoids
Source: Viruses. 2022 Apr 16;14(4):829. doi: 10.3390/v14040829 (PMC9032670; doi:10.3390/v14040829)
Supplement: Supplementary file 1 [file viruses-14-00829-s001.zip › Table S1.pdf]

Supplementary table 1

Supplementary Table 1: Antibodies used in this study for immunofluorescence

Antigen/Target	Host species	Dilutions	Provider, article number
**IBA1**	Goat	1:1000	Abcam, AB5076
**GFAP-pan**	Rabbit	1:1000	Dako, Z0334
**MAP 2**	Rabbit	1:1000	Abcam, AB32454
**GFP**	Chicken	1:1000	Abcam, AB13970
**Chicken, 488**	Donkey	1:1000	Sigma-Aldrich, SAB4600031
**Goat, 568**	Donkey	1:1000	Sigma-Aldrich, SAB4600074
**Rabbit, 568**	Donkey	1:1000	Sigma-Aldrich, SAB4600076
