# Supplementary material for: Characterization of HIV-1 Infection in Microglia-Containing Human Cerebral Organoids
Source: Viruses. 2022 Apr 16;14(4):829. doi: 10.3390/v14040829 (PMC9032670; doi:10.3390/v14040829)
Supplement: Supplementary file 1 [file viruses-14-00829-s001.zip › Table S2.pdf]

Supplementary table 2

Supplementary Table 2: Primer sequences used for qRT-PCR experiments

Gene	5' forward 3'	5' reverse 3'
**CSF1R**	ATCAGCATCCGGCTGAAAGT	CTCGAATCCGCACCAGCTCT
**TREM2**	TCAGGAAGGTCCTGGTGGA	GGGTGGGAAGGGGATTTCTC
**AIF1**	AGACGTTCAGCTACCCTGACTT	GGCCTGTTGGCTTTTCCTTTTCTC
**CX3CR1**	CTTACGATGGCACCCAGTGA	CAAGGCAGTCCAGGAGAGTT
**TMEM119**	CTTCCTGGATGGGATAGTGGAC	GCACAGACGATGAACATCAGC
**P2RY12**	TTTGTGTGTCAAGTTACCTCCG	CTGGTGGTCTTCTGGTAGCG
**GFAP**	AGGTCCATGTGGAGCTTGAC	GCCATTGCCTCATACTGCGT
**ALDH1L1**	GGATGCCTTTGAGAATGGACGG	TCCTGGTGCTGCTCCATGAGAT
**CD4**	TCCAGAGGCTTAATCACACCG	GGCTAGGCTTGAAGGAAAAGG
**CXCR4**	ACTGTTGTCTGAACCCCATCC	AGAGGTGAGTGCGTGCTGG
**Map2**	CTCAGCACCGCTAACAGAGG	CATTGGCGCTTCGGACAAG
**NEUN/RBFOX3**	TTACGGAGCGGTCGTGTATC	CGGGCTGAGCGTATCTGTAG
**TBR2/EOMES**	CGGCCTCTGTGGCTCAAAT	TAGTGGGCAGTGGGATTGAGT
**CCR5**	TTATACATCGGAGCCCTGCC	ATCAGGATGAGGATGACCAGC
**ACTB**	GCTCCTCCTGAGCGCAAG	CATCTGCTGGAAGGTGGACA
