# Supplementary figures and images for: Characterization of HIV-1 Infection in Microglia-Containing Human Cerebral Organoids
Source: Viruses. 2022 Apr 16;14(4):829. doi: 10.3390/v14040829 (PMC9032670; doi:10.3390/v14040829)

# Supplementary Figure S1

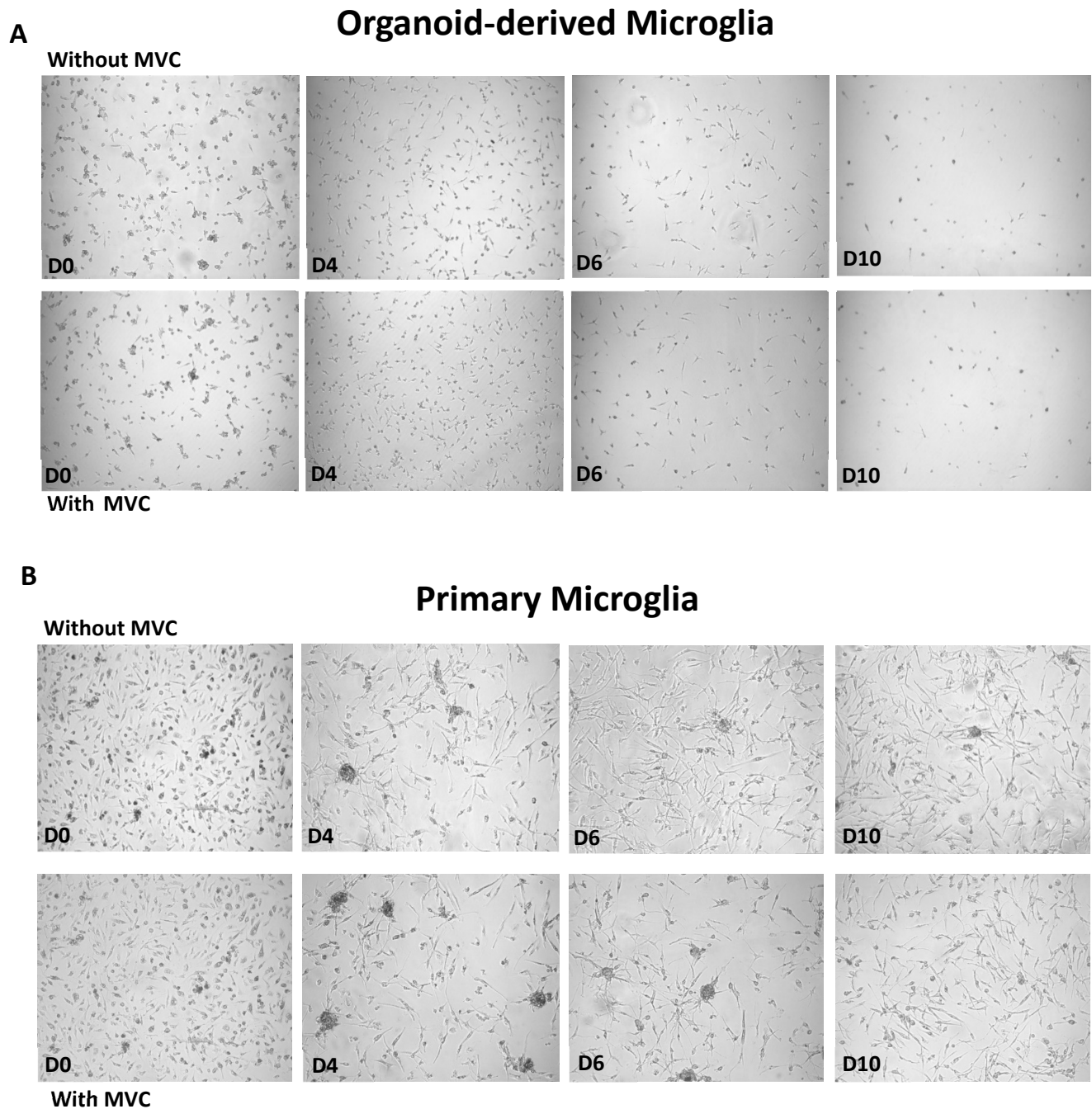

Supplement: Supplementary file 1 [file viruses-14-00829-s001.zip › Figure S1.pdf]

# Supplementary Figure S2

Primary microglia

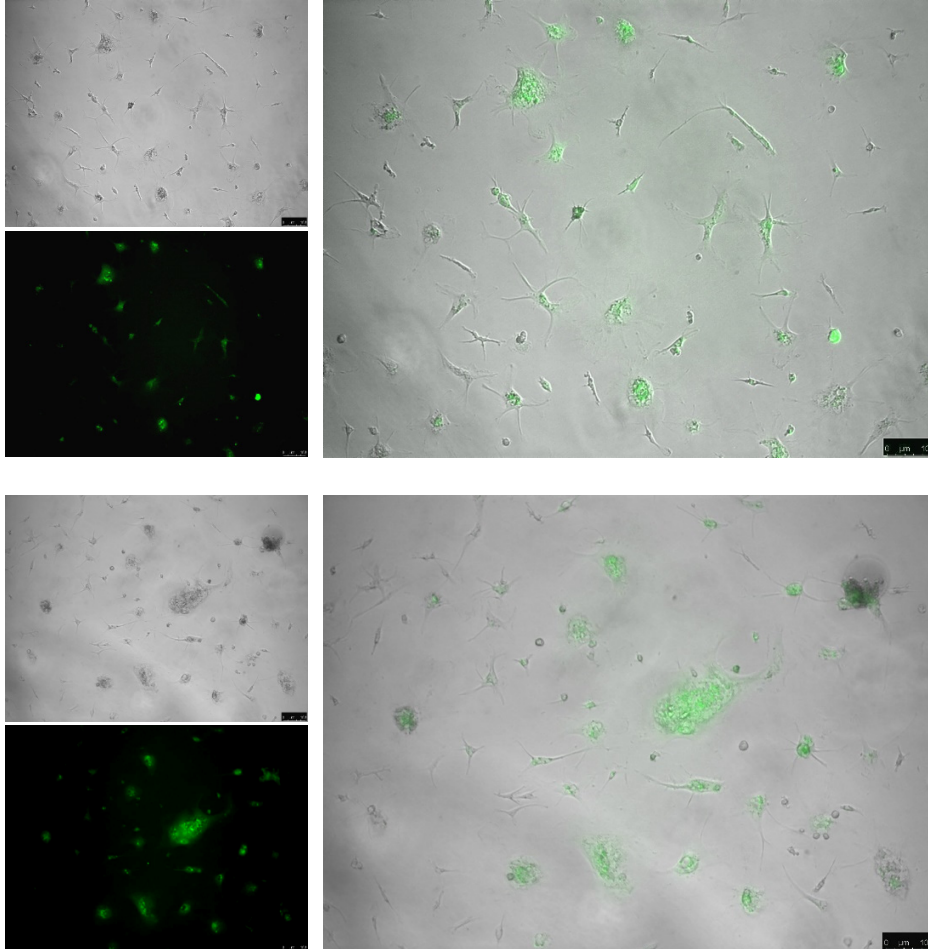

Supplement: Supplementary file 1 [file viruses-14-00829-s001.zip › Figure S2.pdf]

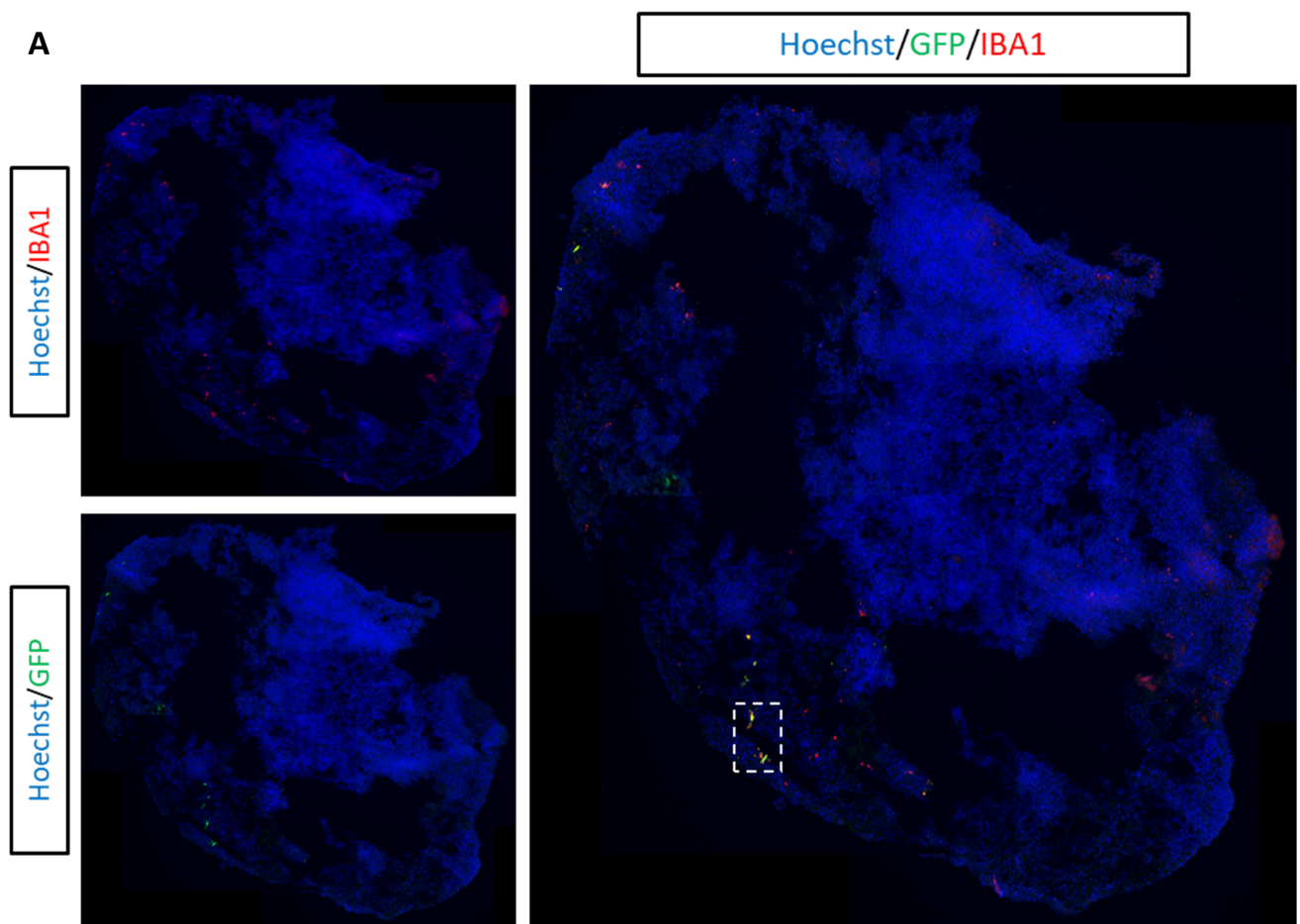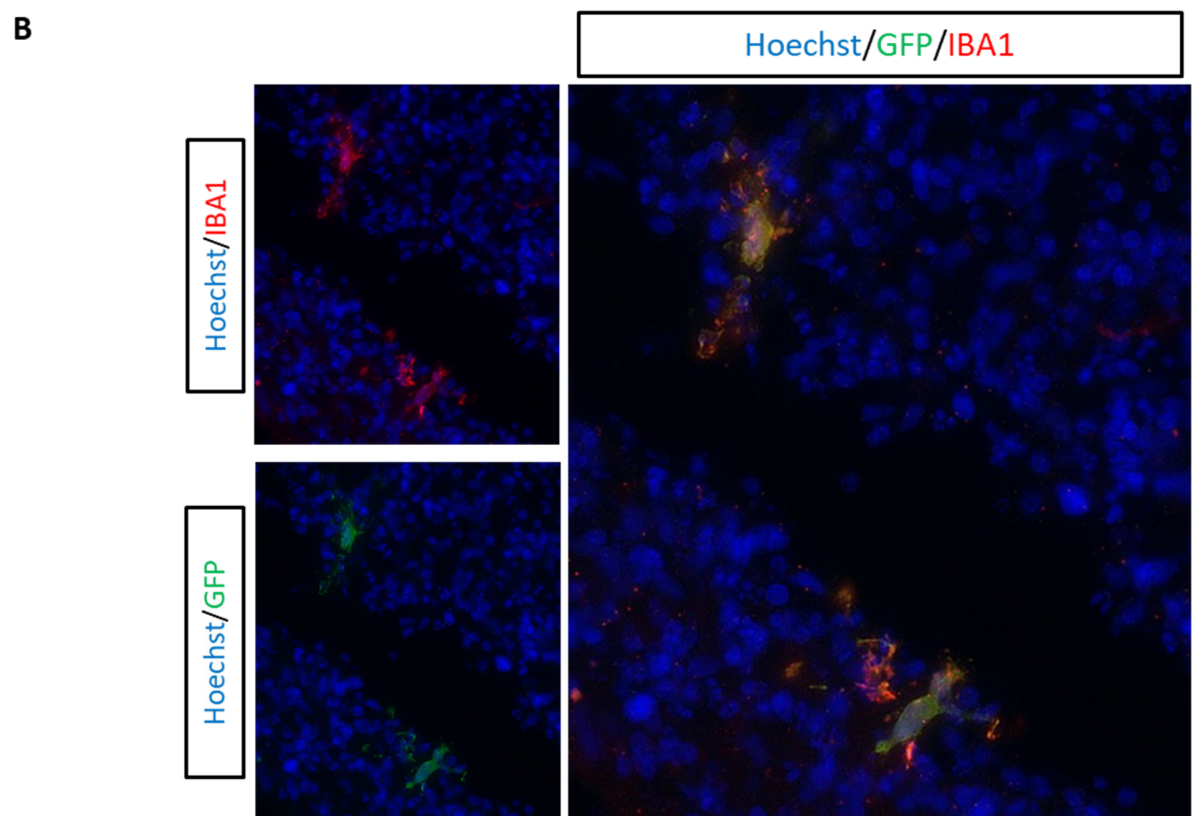

Supplement: Supplementary file 1 [file viruses-14-00829-s001.zip › Figure S3.pdf]

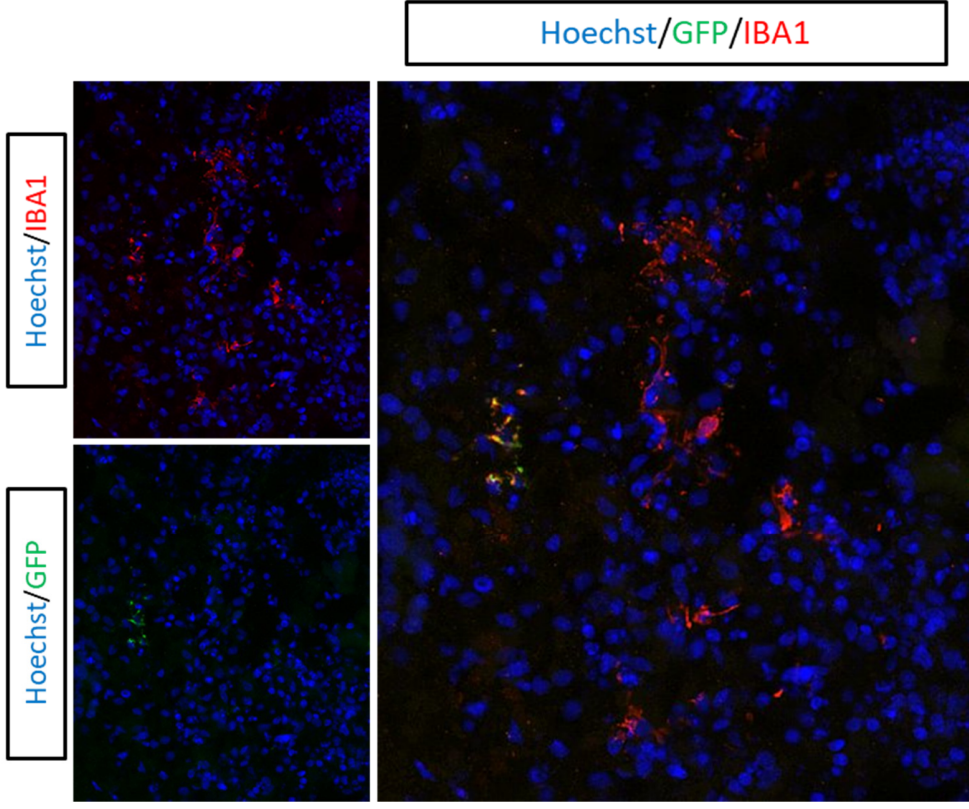

Supplement: Supplementary file 1 [file viruses-14-00829-s001.zip › Figure S4.pdf]
